# Review of published 467 achondroplasia patients: clinical and mutational spectrum

**DOI:** 10.1186/s13023-024-03031-1

**Published:** 2024-01-27

**Authors:** XinZhong Zhang, Shan Jiang, Rui Zhang, Siyi Guo, Qiqi Sheng, Kaili Wang, Yuanyuan Shan, Lin Liao, Jianjun Dong

**Affiliations:** 1 Department of Endocrinology, Qilu Hospital, Cheeloo College of Medicine, Shandong University, Jinan, 250012 China; 2 Department of Endocrinology and Metabology, Shandong Key Laboratory of Rheumatic Disease and Translational Medicine, The First Affiliated Hospital of Shandong First Medical University & Shandong Provincial Qianfoshan Hospital, Shandong Institute of Nephrology, Jinan, China; 3 Department of Endocrinology and Metabology, Shandong Provincial Qianfoshan Hospital, Cheeloo College of Medicine, Shandong University, Jinan, China

**Keywords:** Achondroplasia, *FGFR3*, Molecular study

## Abstract

**Aim:**

Achondroplasia is the most common of the skeletal dysplasias that cause fatal and disabling growth and developmental disorders in children, and is caused by a mutation in the fibroblast growth factor receptor, type 3 gene(*FGFR3*). This study aims to analyse the clinical characteristics and gene mutations of ACH to accurately determine whether a patient has ACH and to raise public awareness of the disease.

**Methods:**

The database of Pubmed, Cochrane Library, Wanfang and CNKI were searched with terms of “Achondroplasias” or “Skeleton-Skin-Brain Syndrome” or “Skeleton Skin Brain Syndrome” or “ACH” and “Receptor, Fibroblast Growth Factor, Type 3” or “*FGFR3*”.

**Results:**

Finally, four hundred and sixty-seven patients with different *FGFR3* mutations were enrolled. Of the 138 patients with available gender information, 55(55/138, 40%) were female and 83(83/138, 60%) were male. Among the patients with available family history, 47(47/385, 12%) had a family history and 338(338/385, 88%) patients were sporadic. The age of the patients ranged from newborn babies to 36 years old. The mean age of their fathers was 37 ± 7 years (range 31–53 years). Patients came from 12 countries and 2 continents, with the majority being Asian (383/432, 89%), followed by European (49/432, 11%). Short stature with shortened arms and legs was found in 112(112/112) patients, the abnormalities of macrocephaly in 94(94/112) patients, frontal bossing in 89(89/112) patients, genu valgum in 64(64/112) patients and trident hand were found in 51(51/112) patients. The most common mutation was *p.Gly380Arg* of the *FGFR3* gene, which contained two different base changes, *c.1138G* > *A* and *c.1138G* > *C*. Ten rare pathogenic mutations were found, including *c.831A* > *C*, *c.1031C* > *G*, *c.1043C* > *G*, *c.375G* > *T*, *c.1133A* > *G*, *c.1130T* > *G, c.833A* > *G*, *c.649A* > *T*, *c.1180A* > *T* and *c.970_971insTCTCCT*.

**Conclusion:**

ACH was caused by *FGFR3* gene mutation, and *c.1138G* > *A* was the most common mutation type. This study demonstrates the feasibility of molecular genetic testing for the early detection of ACH in adolescents with short stature, trident hand, frontal bossing, macrocephaly and genu valgum.

## Introduction

Skeletal dysplasia has been a significant global public health problem. Achondroplasia(ACH, OMIM #100,800) is the most common skeletal dysplasia, its clinical and radiological phenotypes have been described for more than 50 years [1] and occurs in between one in 10,000 and one in 30,000 live births [2].

In 1994, Shiang [3] found that ACH has mutations in the transmembrane domain of the fibroblast growth factor receptor 3 (*FGFR3*) [3] and more than 98% of ACH cases carried the base conversion that changes G to A at position 1138 of cDNA in exon 10 of *FGFR3* gene, which changes the amino acid at position 380 of the *FGFR3* from glycine to arginine. Subsequently, in 1998, Wilkin DJ found that *FGFR3* mutations occur preferentially during spermatogenesis and that the risk of new point mutations increases with the paternal age increasing, while mutations always in paternal alleles in non-familial cases of achondroplasia [4].

The clinical features of achondroplasia are variable, including macrocephaly, brachydactyly, metaphyseal flaring and shortening of the pedicles [5]. The mean height of males was 132 cm and females was 123 cm, which was described by Alderborn in 1996 [5]. Despite the presence of the above clinical manifestations, ACH patients have a natural lifespan and intelligence [6].

The current strategy for identifying patients is to combine the clinical characteristics, imaging findings and molecular genetic testing. With the increasing application of gene sequencing technology, the diagnostic accuracy of ACH has been improved, but the awareness of ACH in public is not enough, which may easily lead to misdiagnosis and missed. Thus, the identification of patients with ACH is great help to give a good birth and good care as soon as possible and it is essential to recognize ACH patients. This study aims to analyse the clinical characteristics and gene mutations of ACH to accurately determine whether a patient has ACH and to raise public awareness of the disease.

## Methods

Pubmed, Cochrane library, the China National Knowledge Infrastructure (CNKI), and Wanfang were searched from the date to 23 March 2023 without language restrictions. The search terms were “Achondroplasias” or “Skeleton-Skin-Brain Syndrome” or “Skeleton Skin Brain Syndrome” or “ACH” and “Receptor, Fibroblast Growth Factor, Type 3” or “*FGFR3*”. Eligible studies met the following criteria: (1) published in English or Chinese; (2) the patients were diagnosed as ACH; (3) the patients confirmed the *FGFR3* mutations by gene diagnosis; and (4) the patients were postnatal.

**Fig. 1:**
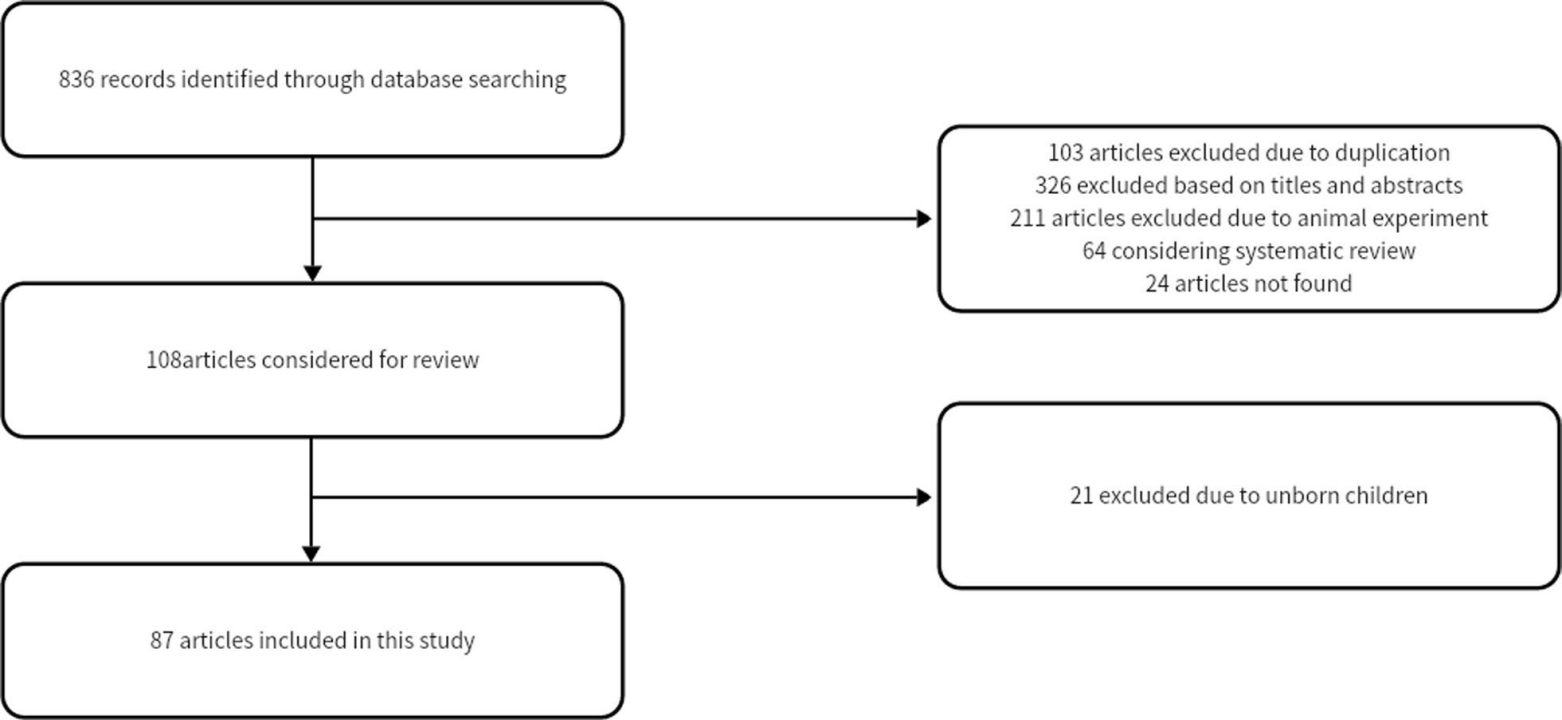
Literature inclusion process

The following clinical characteristics were studied: (1) gender; (2) country; (3) family history; (4) amino acid substitution and type of mutations in the *FRFR3* gene; (5) clinical characteristics. Flow chart of the systematic search process is showed in Fig. 1.

## Results

### Epidemiological characteristics and gene mutations in ACH

Eighty-seven studies including 467 individuals who met the criteria were enrolled. Among them, 432 patients provided the country information. They came from 12 countries and 2 continents, with Asians making up the largest group (383/432, 89%) and Europeans making up 11% (49/432, 11%). Among the Asians, cases from China, Pakistan, Japan, Korea and India accounted for 72%, 11%, 7%, 7%, and 3%, respectively.

The amino acid substitutions and the percentage of mutations in *FGFR3* are listed in Table 1. The most common mutation was *p.Gly380Arg* and 421 patients provided detailed nucleotide changes, of which the proportion of *c.1138G* > *A* was higher than *c.1138G* > *C*, accounting for 97% (410/421, 97%), resulting in the same nucleotide changes, i.e. the glycine was replaced by an arginine. In addition, 6 patients carried the *c.649A* > *T* mutation, 4 patients carried the c*.1180A* > *T* mutation, 3 patients carried the *c.375G* > *T* mutation. The *c.1043C* > *G*, *c.1031C* > *G*, *c.833A* > *G* mutations were all carried by 2 patients, and the *c.831A* > *C* mutations were carried by one patient. Another specific mutation is *c.970_971insTCTCCT.*

**Table 1:** *FGFR3* mutations of ACH patients

References	cDNA	Nucleotide alteration	Protein	Percentage	allele frequencies
[5, 7–84]	c. 1138G > A or c. 1138G > C	glycine to arginine	p.Gly380Arg	95.5% (446/467)	4.79e-6 or 6.85e-7
[14, 89]	c.1031C > G	serine to cysteine	p.Ser344Cys	0.4% (2/467)	NA
[16, 72]	c. 375G > T	glycine to cysteine	p.Gly375Cys	0.6% (3/467)	1.20e-6
[47, 62]	c.833A > G	tyrosine to cysteine	p.Tyr278Cys	0.4% (2/467)	NA
[85]	c.831A > C	serine to cysteine	p.Ser279Cys	0.2% (1/467)	NA
[86]	c.970_971 ins TCTCCT	the insertion of Ser-Phe after position Leu324	p.L324delinsLSF	0.2% (1/467)	NA
[87, 88]	c.1043C > G	serine to cysteine	p.Ser348Cys	0.4% (2/467)	NA
[90, 92]	c.649A > T	serine to cysteine	p.Ser217Cys	1.3% (6/467)	NA
[91]	c.1180A > T	threonine to serine	p.Thr394Ser	0.9% (4/467)	NA

**Fig. 2:**
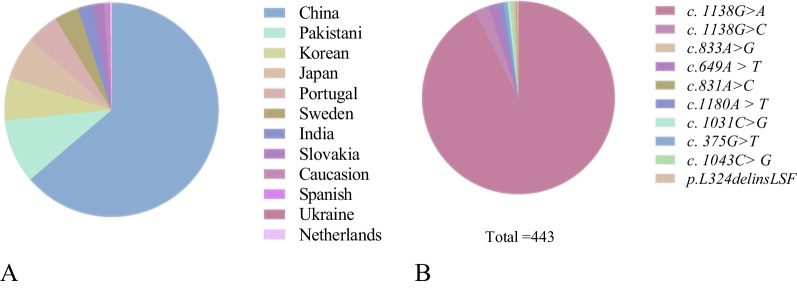
**A** continent distribution radio of patients (%), **B** the percentage of different mutation sites (%)

Most of the patients had one mutation, but 2 patients had two mutations in *FGFR3* on the same allele. One patient carried the common *p.Gly380Arg* mutation and a novel *c.1130T* > *G* mutation [28], and another carried the *p.Gly380Arg* and *c.1133A* > *G *[19]. However, the above two novel mutations had not been reported as the direct pathogenic genes of ACH. Figure 2 shows the detailed information of the enrolled countries and mutation types.

### Clinical characteristics of ACH

Of the 138 patients who provided the gender information, 83(83/138, 60%) were male and 55(55/138, 40%) were female. Of the 385 patients who provided family history information, 47(47/385, 12%) patients had a family history of ACH, 338(338/385, 88%) patients were sporadic.

The age of the patients ranged from newborn babies to 36 years old. Of the 11 and 10 patients who gave the age of their father and mother respectively. The average age of the fathers was 37 ± 7 years old (range from 31 to 53), of which 4(4/11, 36%) were older than 35 years old, and that of the mothers was 32 ± 5 years old (range from 23 to 39).

**Fig. 3:**
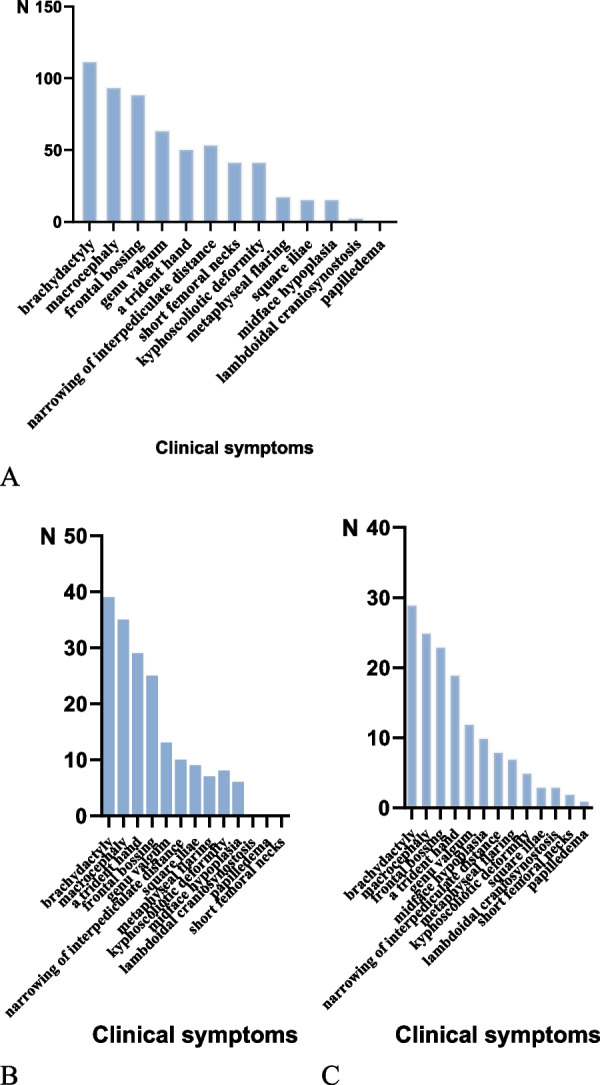
**A** clinical symptoms(N:112), **B** clinical symptoms in male (N:39), **C** clinical symptoms in female (N:29)

A total of 112 patients provided detailed clinical and radiological features, 112(112/112) had short stature with shortened arms and legs, 51(51/112) had the trident hand, 89(89/112) had frontal bossing, 94(94/112) had macrocephaly, 64(64/112) had genu valgum, 54(54/112) had narrowing of the interpediculate distance, 42(42/112) had kyphoscoliotic deformity, 42(42/112) had short femoral necks, 18(18/112) had metaphyseal flaring, 16(16/112) had square iliae and 16(16/112) had midface hypoplasia. Besides the aforementioned manifestations, eleven patients had a history of hydrocephalus found on magnetic resonance imaging. Figure 3 shows details of the clinical symptoms and the differences in the clinical presentation according to gender.

The boy with *c.1130T* > *G* and *c.1138G* > *A* in FGFR3 on the same allele had prolonged episodes of hypoxaemia with respiratory distress and shortness of breath, a chest CT showed pulmonary dysplasia, and a brain MRI showed a very narrow foramen magnum with additional compression of the cervical spine [28]. Similarly, a girl carrying *c.1133G* > *A* and *c.1138G* > *A* in FGFR3 showed more severe clinical and radiological characteristics than classic ACH patients, with respiratory distress, pulmonary hypoplasia, hydrocephalus and cervicomedullary compression [19].

## Discussion

*FGFR3* is located on the short arm of chromosome 4, 4p16.3, and is expressed in chondrocytes and mature osteobl*asts* [93]*.* The main forms of osteogenesis include intramembranous osteogenesis and endochondral osteogenesis, starting with the formation of chondrocytes from mesenchymal cells, followed by the formation of ossification centres from chondrocytes through proliferation and differentiation, and the gradual development of the diaphysis and epiphysis, with chondrocytes located in between promoting linear bone growth through proliferation and differentiation. The activation of *FGFR3* after birth inhibits the proliferation and hypertrophy of chondrocytes [94]. Mutations in *FGFR3* can activate tyrosine protein kinase activity, enhance negative regulatory function, inhibit chondrocyte proliferation, affect bone trabeculae formation, play a role in regulating chondrocyte proliferation and differentiation, and negatively regulate bone growth. In the HGMD database, we found that mutations in the *FGFR3* gene are associated with a variety of diseases, including hypochondroplasia, thanatophoric dysplasia, achondroplasia, craniosynostosis、lacrimo-auriculo-dento-digital syndrome, acanthosis nigricans, prostate cancer and wilms tumour, of which 28 mutations were identified for hypochondroplasia, 15 mutations for thanatophoric dysplasia and 11 mutations for ACH, as shown in Table 2.

**Table 2:** The number of mutations and types in *FGFR3* in HGMD database

Disease/phenotype	Number of mutations
Hypochondroplasia	28
Thanatophoric dysplasia	15
Achondroplasia	11
Craniosynostosis	4
Lacrimo-auriculo-dento-digital syndrome	2
Short stature ?	2
Skeletal dysplasia	2
Acanthosis nigricans	1
Achondroplasia ?	1
Achondroplasia with developmental delay & acanthosis nigricans	1
Achondroplasia with severe Platyspondyly	1
Camptodactyly, tall stature and hearing loss syndrome	1
Cleft lip and palate ?	1
Crouzon syndrome with acanthosis nigricans	1
Prostate cancer	1
Prostate cancer and additional primary cancers	1
Seborrhoeic keratosis ?	1
Short stature	1
Tall stature, lateral tibial deviation, scoliosis, hearing impairment, camptodactyly and arachnodactyly	1
Thanatophoric dysplasia, type 2	1
Wilms tumour	1

Our study demonstrated that 338(338/385, 88%) patients with ACH were sporadic, which was a spontaneous mutation. Four hundred and twenty-one patients provided detailed *p.Gly380Arg* mutations in the *FGFR3* gene, and among them, four hundred and ten patients had *c.1138G* > *A* changes, which was consistent with the studies by Shiang in 1994 [3]. *c.831A* > *C*, *c.1031C* > *G*, *c.1043C* > *G*, *c.375G* > *T*, *c.1133A* > *G*, *c.1130T* > *G*, *c.833A* > *G*, *c.649A* > *T*, *c.1180A* > *T* and *c.970_971insTCTCCT* were ten rare pathogenic mutations. These mutations constitutively activate the *FGFR3* receptor, leading to abnormal membrane ossification, inhibit the growth and proliferation of chondrocytes, and finally hinder the extension of bone. In addition, we also found that there were two novel mutations that occurred simultaneously with the *p.Gly380Arg* mutation, *c.1130T* > *G* and *c.1133A* > *G*. But no related reports indicated that *c.1130T* > *G* and *c.1133A* > *G* were the direct cause of ACH, we did not know whether these mutations were pathogenic or not. Tadashi suggested that these mutations in the same gene may have an additive effect on the activated receptor of the *p.Gly380Arg* mutation and change the protein function, resulting in the severe phenotype of the disease [19].

ACH is an autosomal dominant genetic disorder and the risk of recurrence is associated with whether the parents themselves have ACH. The mean paternal age of the achondroplasia patients analyzed in this study was 37 ± 7 years old(range from 31 to 53), and four of them were over 35 years old. Wilkin analysed 40 families with sporadic ACH and found that the mutated allele was inherited exclusively from the father, suggesting that it affects DNA replication or repair during spermatogenesis [4].

Mutations in the *FGFR3* gene can also cause other types of skeletal dysplasia,, which need to be identified and classified from mild to severe: hypochondroplasia (HCH), achondroplasia, thanodermal dysplasia type I (TD I), severe achondroplasia with developmental delay and nigroschisis (SADDN), and thanodermal dysplasia type II (TD II). HCH is mainly caused by the *c.1620C* > *A* or *c.1651A* > *G* mutations, and patients usually present with mid-craniofacial deformities, limb deformities, and hand and foot deformities. In contrast, TD has more severe clinical manifestations than ACH, which can be divided into TD I and TD II. TD I is caused by the *c.742C* > *T*, *c.1111A* > *T* and *c.1118A* > *G* mutations in *FGFR3*, and TD II is mainly caused by the *c.1948A* > *G* mutation. SADDAN syndrome is a severe form of ACH associated with growth retardation and acanthosis nigricans caused by the *c.1949 A* > *T* mutation. In addition, previous studies have shown that skeletal abnormalities and growth disorders are associated with defects in the *SHOX* gene, such as Leri-Weill syndrome (LWD), Turner syndrome (TS) and idiopathic short stature (ISS). The *SHOX* gene is located at the end of the short arms of the X and Y sex chromosomes (Xp22.32 or Ypll.3) and was first identified in 1997 by Rao et al. [95]. Early detection of *SHOX* gene mutations and skeletal malformations is an important guideline for the diagnosis and management of dwarfism. Common clinical manifestations include short forearm and lower leg, cubitus valgus, Madelung deformity, high-arched palate and muscular hypertrophy [96]. In this study, we summarized the *FGFR3* mutation types of eighty-seven studies including 467 individuals and the clinical characteristics of 112 patients with ACH. Some common clinical characteristics of ACH were as follows: (1) short stature with shortened arms and legs (112/112); (2) trident hand (51/112); (3) frontal bossing (89/112); (4) macrocephaly (94/112); (5) genu valgum (64/112). The following radiological characteristics were common: (1) the narrowing of the interpediculate distance (54/112); (2) kyphoscoliotic deformity (42/112); (3) short femoral necks (42/112).

Based on the main clinical features (short stature, macrocephaly, frontal bossing, midface hypoplasia, genu valgum) and radiological features (square iliae, narrowing of interpediculate distance, kyphoscoliotic deformity, short femoral necks) can be diagnosed clinically in most patients with ACH. In patients with clinical or radiological suspicion of ACH, it would be easy to determine the two most common pathogenic variants of the *FGFR3* mutation in the affected child by PCR. For children without mutations at common mutation sites or requiring differential diagnosis of ACH, whole-exon *FGFR3* sequencing should be used for detection. Prenatal screening programmes for ACH usually include chorionic villus sampling, amniocentesis and ultrasound. The realisation of early diagnosis and early treatment not only has a good therapeutic effect, but also reduces the burden of the disease and saves on the cost of medical care.

So far, there is no standardized treatment for ACH in the world. At present, the treatment of ACH mainly includes symptomatic treatment and surgical intervention. In this study, four patients were received growth hormone treatment, three of whom had an increase in height after six months, one with growth hormone 0.15 U/kg per day alone and two with growth hormone 2.5IU per day combined with L-thyroid hormone 12.5ug per day. Among them, two patients described the accurate figures of the increase, which were 8cm and 3.8 cm, respectively. Two patients received L-thyroxine while taking growth hormone, and all of them gained height growth. However, the sample data are too small to conclude whether L-thyroxine could promote the effect of growth hormone and we also could not get the right dose of growth hormone and the right treatment cycle. In 2005, Hertelt treated 35 pre-adolescent ACH children with recombinant human growth hormone (rhGH) 0.1 IU/kg or 0.2 IU/kg per day for 5 years, and found that the average growth rate increased significantly by 1.9/3.6 cm/year in the first year and 0.5/1.5 cm/year in the second year [97]. The short-term effect of rhGH on the height growth of ACH may be ideal. The growth of height and bone age of untreated ACH children are increasingly lagging behind that of children matched on age and sex [97]. Growth hormone is an important positive regulator of linear bone growth and promotes epiphyseal growth in children by stimulating hepatic production of insulin-like growth factor-1, which promotes chondrocyte growth and metabolism. One drug currently in development for the treatment of ACH is C-type natriuretic peptide (CNP), the overexpression of which in cartilage tissue is protective against chondrodysplasia [98], e.g. vasoretin. Binding of vosolide to NPR-B stimulates intracellular cyclic guanosine monophosphate (cGMP) production, which in turn inhibits the downstream signalling pathway of *FGFR3* and promotes chondrocyte proliferation, differentiation and endochondral bone formation and has been proved to restore normal bone growth in a mouse model of ACH. Several clinical trials have shown that the annual growth rate of patients with ACH has increased after treatment with the vosoritide, and no significant adverse effects were observed [99–101]. ACH can be treated surgically by limb lengthening, but high risk of postoperative complications still exists [102].

Our study has several limitations. First, in view of there were few articles that explicitly mentioned the country, the countries included in the article mainly included Asian countries such as China, Japan and Korea, but few countries in Europe and other continents. Second, 24 articles failed to find the full text and were excluded. Third, the included articles contained few treatment methods, so that we could not get the appropriate treatment scheme.

At present, there are obvious global differences in the clinical treatment of patients with achondroplasia. This variability leads to different results on the medical, functional and psychological consequences of achondroplasia. Exercise intolerance and exercise-induced fatigue are common symptoms in children with achondroplasia. The physical performance and the muscle strength of children with achondroplasia are weakened compared with that of general population [103]. The difference in body structure of ACH patients may lead directly or indirectly lead to the limitations in activity and participation, including interpersonal communication, physical performance and self-care. ACH is the most common bone dysplasia, which faces various medical and psychosocial challenges in life. We should promote the improvement and standardization of nursing methods, realize multidisciplinary management in the whole life cycle, and optimize its clinical outcome and life quality.

## Data Availability

All data extracted from the included studies are publicly available in PubMed (https://pubmed.ncbi.nlm.nih.gov/), Cochrane (https://www.cochrane.org/), CNKI (https://www.cnki.net/) and WanFang (https://g.wanfangdata.com.cn/).

## References

[1] Cohen MM (1998). Achondroplasia, hypochondroplasia and thanatophoric dysplasia: clinically related skeletal dysplasias that are also related at the molecular level. Int J Oral Maxillofac Surg.

[2] Waller DK, Correa A, Vo TM (2008). The population-based prevalence of achondroplasia and thanatophoric dysplasia in selected regions of the US. Am J Med Genet A.

[3] Shiang R, Thompson LM, Zhu YZ, Church DM, Fielder TJ, Bocian M, Winokur ST, Wasmuth JJ (1994). Mutations in the transmembrane domain of FGFR3 cause the most common genetic form of dwarfism, achondroplasia. Cell.

[4] Wilkin DJ, Szabo JK, Cameron R (1998). Mutations in fibroblast growth-factor receptor 3 in sporadic cases of achondroplasia occur exclusively on the paternally derived chromosome. Am J Hum Genet.

[5] Alderborn A, Anvret M, Gustavson KH, Hagenäs L, Wadelius C (1996). Achondroplasia in Sweden caused by the G1138A mutation in FGFR3. Acta Paediatr.

[6] Kale L, Khambete N, Sodhi S, Kumar R (2013). Achondroplasia with oligodontia: report of a rare case. J Oral Maxillofac Pathol.

[7] Henderson S, Sillence D, Loughlin J, Bennetts B, Sykes B (2000). Germline and somatic mosaicism in achondroplasia. J Med Genet.

[8] Wright MJ, Ain MC, Clough MV, Bellus GA, Hurko O, McIntosh I (2000). Achondroplasia and nail-patella syndrome: the compound phenotype. J Med Genet.

[9] Yan-Ling G, Ji-Hong N, Guo-Qiang L, Wei W, De-Fen W (1998). FGFR3 gene mutations in transmembrane domain in Chinese achondroplasia and hypochondroplasia patients. Horm Res.

[10] Ajmal M, Mir A, Shoaib M, Malik SA, Nasir M (2017). Identification and in silico characterization of pG380R substitution in FGFR3, associated with achondroplasia in a non-consanguineous Pakistani family. Diagn Pathol.

[11] Almeida MR, Campos-Xavier AB, Medeira A, Cordeiro I, Sousa AB, Lima M, Soares G, Rocha M, Saraiva J, Ramos L, Sousa S, Marcelino JP, Correia A, Santos HG (2009). Clinical and molecular diagnosis of the skeletal dysplasias associated with mutations in the gene encoding fibroblast growth factor receptor 3 (FGFR3) in Portugal. Clin Genet.

[12] Bessenyei B, Nagy A, Balogh E, Novák L, Bognár L, Knegt AC, Oláh E (2013). Achondroplasia with multiple-suture craniosynostosis: a report of a new case of this rare association. Am J Med Genet A.

[13] Ceroni JRM, Soares DCQ, Testai LC, Kawahira RSH, Yamamoto GL, Sugayama SMM, Oliveira LAN, Bertola DR, Kim CA (2018). Natural history of 39 patients with Achondroplasia. Clinics (Sao Paulo).

[14] Chang IJ, Sun A, Bouchard ML, Kamps SE, Hale S, Done S, Goldberg MJ, Glass IA (2018). Novel phenotype of achondroplasia due to biallelic FGFR3 pathogenic variants. Am J Med Genet A.

[15] Georgoulis G, Alexiou G, Prodromou N (2011). Achondroplasia with synostosis of multiple sutures. Am J Med Genet A.

[16] Ikegawa S, Fukushima Y, Isomura M, Takada F, Nakamura Y (1995). Mutations of the fibroblast growth factor receptor-3 gene in one familial and six sporadic cases of achondroplasia in Japanese patients. Hum Genet.

[17] Kotysova L, Mattosova S, Chandoga J (2015). Improvement of molecular-genetic diagnostics of the most common skeletal dysplasias. Bratisl Lek Listy.

[18] Po Z, Hongwei MA, Yang W, Zhen M, Yingyu WU, Mei J, Hong GAO, Yongxin L (1999). Mutations of the fibroblast growth factor receptor 3 gene in achondroplasia. Chin J Med Genet.

[19] Nagata T, Matsushita M, Mishima K, Kamiya Y, Kato K, Toyama M, Ogi T, Ishiguro N, Kitoh H (2020). Severe achondroplasia due to two de novo variants in the transmembrane domain of FGFR3 on the same allele: A case report. Mol Genet Genomic Med.

[20] Nastasi S, Gonzalez A, Blake CR, Beck A, Agarwal-Sinha S (2018). Achondroplasia With Congenital Onset Glaucoma, and Presumed Axenfeld-Rieger Anomaly. J Glaucoma.

[21] Jihong N, Guoqiang LU, Wei W, Fengsheng C, Huili Q, Defen W (2002). Detection of fibroblast growth factor receptor 3 gene mutation at nucleotide 1138 site in congenital achondroplasia patients. Chin J Med Genet.

[22] Numakura C, Kobayashi H, Hasegawa Y, Adachi M, Kim OH, Nishimura G (2007). Achondroplasia and enchondromatosis: report of three boys. Skeletal Radiol.

[23] Patil SJ, Banerjee M, Phadke SR, Mittal B (2009). Mutation analysis in Indian children with achondroplasia - utility of molecular diagnosis. Indian J Pediatr.

[24] Pugash D, Lehman AM, Langlois S (2014). Prenatal ultrasound and MRI findings of temporal and occipital lobe dysplasia in a twin with achondroplasia. Ultrasound Obstet Gynecol.

[25] Ros-Pérez P, Regidor FJ, Colino E, Martínez-Payo C, Barroso E, Heath KE (2012). Achondroplasia with 47, XXY karyotype: a case report of the neonatal diagnosis of an extremely unusual association. BMC Pediatr.

[26] Ross JL, Bellus G, Scott CI, Abboudi J, Grigelioniene G, Zinn AR (2003). Mesomelic and rhizomelic short stature: the phenotype of combined Leri-Weill dyschondrosteosis and achondroplasia or hypochondroplasia. Am J Med Genet A.

[27] Rousseau F, Bonaventure J, Legeai-Mallet L, Pelet A, Rozet JM, Maroteaux P, Le Merrer M, Munnich A (1994). Mutations in the gene encoding fibroblast growth factor receptor-3 in achondroplasia. Nature.

[28] Rump P, Letteboer TG, Gille JJ, Torringa MJ, Baerts W, van Gestel JP, Verheij JB, van Essen AJ (2006). Severe complications in a child with achondroplasia and two FGFR3 mutations on the same allele. Am J Med Genet A.

[29] Shin YL, Choi JH, Kim GH, Yoo HW (2005). Comparison of clinical, radiological and molecular findings in Korean infants and children with achondroplasia and hypochondroplasia. J Pediatr Endocrinol Metab.

[30] Sobetzko D, Braga S, Rüdeberg A, Superti-Furga A (2000). Achondroplasia with the FGFR3 1138g–>a (G380R) mutation in two sibs sharing a 4p haplotype derived from their unaffected father. J Med Genet.

[31] Tonoki H, Nakae J, Tajima T, Shinohara N, Monji J, Satoh S, Fujieda K (1995). Predominance of the mutation at 1138 of the cDNA for the fibroblast growth factor receptor 3 in Japanese patients with achondroplasia. Jpn J Hum Genet.

[32] Yang SW, Kitoh H, Yamada Y, Goto H, Ogasawara N (1998). Mutation in the gene encoding the fibroblast growth factor receptor-3 in Korean children with achondroplasia. Acta Paediatr Jpn.

[33] Yuan H, Huang L, Hu X, Li Q, Sun X, Xie Y, Kong S, Wang X (2016). FGFR3 gene mutation plus GRB10 gene duplication in a patient with achondroplasia plus growth delay with prenatal onset. Orphanet J Rare Dis.

[34] Ye Z, Weimin YU, Ming S, Qing F, Muzhen F (2000). Differentiation of achondroplasia and other similar genetic dwarfism by FGFR3 gene analysis [J]. Chin J Med Genet.

[35] Haiyan Z, Ying Y, Jie L, Tong R, Yari HU (2008). Rapid genetic prenatal diagnosis for achondroplasia [J]. Chin J Obstetr Gynecol.

[36] Arditi JD, Thomaidis L, Frysira H, Doulgeraki A, Chrousos GP, Kanaka-Gantenbein C (2017). Long-term follow-up of a child with Klinefelter syndrome and achondroplasia from infancy to 16 years. J Pediatr Endocrinol Metab.

[37] Chen H, Mu X, Sonoda T, Kim KC, Dailey K, Martinez J, Tuck-Muller C, Wertelecki W (2000). FGFR3 gene mutation (Gly380Arg) with achondroplasia and i(21q) Down syndrome: phenotype-genotype correlation. South Med J.

[38] Yan-mei H, Li-wei G, Duan LI, Ying-jie QI, Bao-sheng Y (2009). Detecting and analyzing on mutation of fibroblost growth factor recepter 3 gene in a congenital achondroplasia family [J]. Journal of Practical Pediatrics.

[39] Clinical analysis in 44 patients with short stature 2. Mutation study of FGFR3 gene in 3 achondroplasia families[D]. Inner Mongolia Medical University,2013 (in Chinese)

[40] Shuli H, The hip fracture incidence inBeijing area, China, during the year 2002 to 2006 2.G1138A mutation in FGFR3 gene causes achondroplasia [D]. China Union Medical College,2010. (in Chinese)

[41] Bai B, Xin YP, Tian GE (2015). A case report of achondroplasia syndrome [J]. Chin J Disab Med.

[42] Xiaoli Y, Yanqin LU, Yunzhang D, Yao Z, Xiang M, Jinxiang H (2018). Detection FGFR3 gene mutation in four achondroplasia patients[J]. J Rare Dis.

[43] Shuli H, Weibo X, Yue S (2010). G1138A mutation in FGFR3 gene causes achondroplasia. Chin J Pract Med.

[44] Limei A, Weiwei LI, Ke LI, Jiehua MA, Xinyi X, Yingxia C, Yufeng H (2012). Prenatal molecular diagnosis of achondroplasia caused by FGFR3 gene mutation. Chin J Birth Health Heredity.

[45] Hongwei M, Jun J, Junfeng L, Guohui N, Yao L, Liping L, Yang Y, Ailu C, Tao S, Hui L (2005). Application of FGFR3 gene mutation analysis in prenatal diagnosis and fetus with brachymelia [J]. Chin J Pract Pediatr.

[46] Xu J. Research on Clinical and Molecular Etiology of Monogenic Hereditary Bone Disease Abstract [D]. Soochow University,2014. (in Chinese)

[47] Fang L, Hongwei M, Ying S et al. Clinical analysis and genetic diagnosis of short-limb inherited short stature diseases in children[C]. In: Proceedings of the 18th National Pediatric Academic Conference of the Chinese Medical Association. 2013:285–285. (in Chinese)

[48] Zhancheng L, Ying C, Lijuan W, Bin Z, Jin-sheng G (2003). Differential diagnosis of achondroplasia by FGFR3gene analysis[J]. Chin J Eugen Genet.

[49] Liang X, Hanmin L, Meng M, Li D (2018). Mutation analysion FGFR3 gene in two Chinese achondroplasia pedigrees [J]. J Chengdu Med Coll.

[50] Libin M Genetic etiology analysis of Madelung malformation family, congenital chondrodysplasia family, and Nori disease family [D]. Central South University, 2012 (in Chinese)

[51] Yang Li Gu, Qiang PJ, Jun Z, Jing X (2013). Gene mutation detection in a patient with achondroplas [J]. Int J Genet.

[52] Yamei Z, Naijun W, Huihui S (2018). Clinical features and FGFR3 gene of a patient with achondroplasia [J]. Armed Police Medicine.

[53] Po Z, Hongwei Ma, Yang W, Mi Zhen Wu, Yingyu JM, Hong G (1999). Mutations of the fibroblast growth factor receiver 3 gene in achondroplasia [J]. Chin J Med Genet.

[54] Fujun Z, Qingyang C, Xinyang Z, Qianfang J, Shasha Li, Jiaxiu Z (2020). Clinical and gene variation analysis of 2 cases of achondroplasia replicated by global developmental delay [J]. Chin J Pract Pediatr Clin.

[55] Yu L, Ying X, Lu C, Tingting S, Fenfen G, Hui X, Jianfang Z (2017). The gene sequencing and preliminary diagnosis of achondroplasia [J]. Chin J Eugen Genet.

[56] Ming S, Chongning, Peichang L, Weimin Y, Shuxiang J (1999). Gene diagnosis and management of a case of chondrodysplasia combined with hydrocephalus [J]. Beijing Med J.

[57] Hongwei Ma, Shijun Ji, Po Z, Hong G, Yang W, Mi Zhen Wu, Yingyu.  (1999). Mutation of fibroclast growth factor receiver 3 gene and achondroplasia [J]. Chin J Pediatr Surg.

[58] Jingfang L, Xulei T, Jianguo C, Liting W, Xiaomei Y, Yan W (2014). Student on clinical characteristics and molecular mechanisms of an achondroplasia child [J]. J Shanxi Med Univ.

[59] Shen Ming Yu, Weimin ZS, Jun G, Muzhen F, Ning P, Peichang L, Xuezhe Z (1997). Detection of FGFR3 gene mutation in achondroplasia [J]. Chin J Pediatr.

[60] Xinyi X, Weiwei L, Qiuyue W, Na L, Yang Z, Yingxia C, Xiaojun L, Jinsheng Z (2013). FGF receptor gene screening in three achondroplasia patients. Chin J Eugen Gen.

[61] Yao W, Yaya C, Xiaoyan Z, Jinxiang H (2014). Mutations analysis of the fibroblast growth factor receiver 3 gene in achondroplasia [J]. J Rare Rare Dis.

[62] Nan S, Yangli X, Can L, Lin C FGFR3 gene mutations in patients with chondrodysplasia [C]//. Summary of the 2012 National Conference on Developmental Biology [Publisher unknown], 2012:152–153 (in Chinese)

[63] Jing He, Chen Hong Su, Jie LP, Yinhong Z, Chanchan J, Shengli Z, Baosheng Z (2014). Genetic diagnosis of fibroblast growth factor receptor 3 gene in achondroplasia patients [J]. J Clin Lab.

[64] Yuanyuan L, Yan J, Yanfang H, Wang Ou, Mei Li, Xiaoping X, Jin D, Weibo X (2015). Clinical features and FGFR3 gene mutation of patients with achondroplasia [J]. Chin J Osteopor Bone Miner Dis.

[65] Qian P, Zhang Yu, Li Jing Wu, Qing YY (2008). Mutation detection of FGFR3 gene and preliminary diagnosis of an achondroplasia family [J]. J Clin Pediatr.

[66] Ning L, Huirong S, Qinghua W, Miao J, Xiangdong K (2013). Mutation analysis of FGFR3 gene and preliminary diagnosis in the first trimester of pregnancy of an achondroplasia family [J]. Chin J Eugen Genet.

[67] Jia X, Qinglin K, Zeng Z, Jinwei H, Liansong L, Wenzhen F, Zhenlin Z FGFR3 gene mutation detection and literature review in a family with chondrodysplasia [C]//. Compilation of papers from the 11th National Endocrinology Academic Conference of the Chinese Medical Association [Publisher unknown], 2012:365 (in Chinese)

[68] Longjiang Z, Wei C, Xia L, Qin Z, Xiu Z (2015). Study on G1138A mutation of fibroblast growth factor receptor 3 gene in a family with chondrodysplasia [J]. Chin J Pract Pediatr Clin Pract.

[69] Jingfang L, Xulei T, Jianguo C, Liting W, Xiaomei Y, Yan W Clinical and FGFR3 gene mutations in a family with chondrodysplasia [C]//. Compilation of papers from the 12th National Endocrinology Academic Conference of the Chinese Medical Association [Publisher unknown], 2013:577 (in Chinese)

[70] Lindi Li, Dan L, Yang Hu, Tiantian Xu, Qiongyan Li, Zongyan G (2014). FGFR3 gene mutation analysis of achondroplasia and hypocondroplasia families [J]. J Clin Pediatr.

[71] Lindi L Clinical presentations and gene analysis of dyschordoplasia and chromosome 22q11 microdeletion syndrome [D]. Guangxi Medical University, 2015 (in Chinese)

[72] Renan W, Xudong C, Yuzhuan Z (2020). Clinical characteristics and FGFR3 gene mutation analysis about 7 cases of patients with achondroplasia in Sanya area [J]. Chin J Eugen Gen.

[73] Yibin G, Hongda P, Chunmiao G, Jingxin P, Zhao Yan Du, Chuanshu.  (2010). Rapid detection of FGFR3 gene mutation super hot spot with double mismatch base Pairs ARMS combined with RE assist [J]. J Mol Diagn Treat.

[74] Zhou X. Genetic diagnosis and prenatal diagnosis of four hereditary bone diseases [D]. Nanjing Normal University, 2011. (in Chinese)

[75] Yan J, Yue G (2019). Cartilage dysplasia congenital hydrocephalus merger case analysis [J]. Chin J Healthy Birth Hered.

[76] Jihong N, Guoqiang L, Wei W, Fengsheng C, Defen W Detection of 1138 nucleotide point mutation of FGFR3 gene in congenital achondroplasia [C]//. A compilation of papers of the Sixth National Endocrinology Conference of Chinese Medical Association.[Publisher unknown],2001:101. (in Chinese)

[77] Ni Jihong Lu, Guoqiang WW, Fengsheng C, Huili Q, Defen W (2002). Detection of fibroblast growth factor receptor 3 gene mutation at nucleotide 1138 site in congenital achondroplasia patients[J]. Chin J Med Genet.

[78] Gao Jing, Wei Haiyan. Clinical characteristics and gene detection analysis of congenital achondroplasia [C]//. Compilation of papers of the 15th National Academic Conference of Medical Genetics of the Chinese Medical Association and the First National Academic Conference of the Medical Geneticist Branch of the Chinese Medical Doctor Association and the 2016 Annual Conference of Medical Genetics of Zhejiang Province.[Publisher unknown],2016:111. (in Chinese)

[79] Na Z, Weiqing W, Lei J, Lei Y, Wenqiang F, Yufang B, Liqing G, Yongji Z, Guang N (2004). Genetical diagnosis in a congenital achondroplasia family[J]. Chin J Endocrinol Metabol.

[80] Yuxiang L, Aizhen Y, Xiaoli L, Fenghua L Molecular diagnosis and prenatal molecular diagnosis of a family with congenital achondroplasia [C]//. Biological engineering society of China in 2014, the annual meeting and the academic conference proceedings of biotechnology. [publisher unknown], 2014–60. DOI: 10.26914 / Arthur c. nkihy. 2014.001969. (in Chinese)

[81] Junjiang Fu, Luyun L, Guangxiu L (2001). Rapid detection of FGFR3 gene mutations in a patient with achondroplasia [J]. Chin J Med Gen.

[82] Hua L, Mingyi MA, Ting D, Wei Q, Juan L, Yuxiang Z (2013). Mutation detection of FGFR3 gene and prenatal diagnosis of an achondroplasia sporadic family[J]. Chin J Modern Med.

[83] Hongwei M, Wang, Mei J, Po Z, Zhen M, Hong G (1999). A simple and rapid method for detecting G380R mutation of FGFR3 gene in achondroplasia [J]. J China Med Univ.

[84] Shan L, Han W, Hua S, Jinsong G, Xiuli Z (2017). Rapid detection of hot spot mutations of FGFR3 gene with PCR-high resolution melting assay[J]. Chin J Med Gen.

[85] Heuertz S, Le Merrer M, Zabel B, Wright M, Legeai-Mallet L, Cormier-Daire V, Gibbs L, Bonaventure J (2006). Novel FGFR3 mutations creating cysteine residues in the extracellular domain of the receptor cause achondroplasia or severe forms of hypochondroplasia. Eur J Hum Genet.

[86] Meyer AN, Modaff P, Wang CG, Wohler E, Sobreira NL, Donoghue DJ, Pauli RM (2021). Typical achondroplasia secondary to a unique insertional variant of FGFR3 with in vitro demonstration of its effect on FGFR3 function. Am J Med Genet A.

[87] Chaudhry C, Srivastava P, Das R, Kaur J, Panigrahi I, Kaur A (2021). Achondroplasia-first report from india of a rare FGFR3 gene variant. Lab Med.

[88] Hasegawa K, Fukuhara R, Moriwake T, Tanaka H, Higuchi Y, Yamashita M, Tsukahara H (2016). A novel mutation p.Ser348Cys in FGFR3 causes achondroplasia. Am J Med Genet A.

[89] Takagi M, Kouwaki M, Kawase K, Shinohara H, Hasegawa Y, Yamada T, Fujiwara I, Sawai H, Nishimura G, Hasegawa T (2015). A novel mutation Ser344Cys in FGFR3 causes achondroplasia with severe platyspondyly. Am J Med Genet A.

[90] Zhang SR, Zhou XQ, Ren X, Wang TT, Yuan MX, Wang Q, Liu JY, Liu MG (2007). Ser217Cys mutation in the Ig II domain of FGFR3 in a Chinese family with autosomal dominant achondroplasia. Chin Med J (Engl).

[91] Bin Z, Qiuming D, Xinghua H, Guoqing J, Ying C, Wenxing W, Haiyan J, Jinsheng G (2003). Mutation analysis of fibroblast growth factor receptor 3 gene in an achondroplasia family[J]. Chin J Med Gen.

[92] Zhang SR Analysis of Achondroplasia and Congenital Cataract in Families[D]. Huazhong University of Science and Technology,2007. (in Chinese)

[93] Kelleher FC, O'Sullivan H, Smyth E, McDermott R, Viterbo A (2013). Fibroblast growth factor receptors, developmental corruption and malignant disease. Carcinogenesis.

[94] Karuppaiah K, Yu K, Lim J, Chen J, Smith C, Long F, Ornitz DM (2016). FGF signaling in the osteoprogenitor lineage non-autonomously regulates postnatal chondrocyte proliferation and skeletal growth. Development.

[95] Rao E, Weiss B, Fukami M, Rump A, Niesler B, Mertz A, Muroya K, Binder G, Kirsch S, Winkelmann M, Nordsiek G, Heinrich U, Breuning MH, Ranke MB, Rosenthal A, Ogata T, Rappold GA (1997). Pseudoautosomal deletions encompassing a novel homeobox gene cause growth failure in idiopathic short stature and Turner syndrome. Nat Genet.

[96] Rappold G, Blum WF, Shavrikova EP, Crowe BJ, Roeth R, Quigley CA, Ross JL, Niesler B (2007). Genotypes and phenotypes in children with short stature: clinical indicators of SHOX haploinsufficiency. J Med Genet.

[97] Hertel NT, Eklöf O, Ivarsson S, Aronson S, Westphal O, Sipilä I, Kaitila I, Bland J, Veimo D, Müller J, Mohnike K, Neumeyer L, Ritzen M, Hagenäs L (2005). Growth hormone treatment in 35 prepubertal children with achondroplasia: a five-year dose-response trial. Acta Paediatr.

[98] Krejci P, Masri B, Fontaine V, Mekikian PB, Weis M, Prats H, Wilcox WR (2005). Interaction of fibroblast growth factor and C-natriuretic peptide signaling in regulation of chondrocyte proliferation and extracellular matrix homeostasis. J Cell Sci.

[99] Savarirayan R, Tofts L, Irving M, Wilcox WR, Bacino CA, Hoover-Fong J, Font RU, Harmatz P, Rutsch F, Bober MB, Polgreen LE, Ginebreda I, Mohnike K, Charrow J, Hoernschemeyer D, Ozono K, Alanay Y, Arundel P, Kotani Y, Yasui N, White KK, Saal HM, Leiva-Gea A, Luna-González F, Mochizuki H, Basel D, Porco DM, Jayaram K, Fisheleva E, Huntsman-Labed A, Day JRS (2021). Safe and persistent growth-promoting effects of vosoritide in children with achondroplasia: 2-year results from an open-label, phase 3 extension study. Genet Med.

[100] Savarirayan R, Tofts L, Irving M, Wilcox W, Bacino CA, Hoover-Fong J, Ullot Font R, Harmatz P, Rutsch F, Bober MB, Polgreen LE, Ginebreda I, Mohnike K, Charrow J, Hoernschemeyer D, Ozono K, Alanay Y, Arundel P, Kagami S, Yasui N, White KK, Saal HM, Leiva-Gea A, Luna-González F, Mochizuki H, Basel D, Porco DM, Jayaram K, Fisheleva E, Huntsman-Labed A, Day J (2020). Once-daily, subcutaneous vosoritide therapy in children with achondroplasia: a randomised, double-blind, phase 3, placebo-controlled, multicentre trial. Lancet.

[101] Chan ML, Qi Y, Larimore K, Cherukuri A, Seid L, Jayaram K, Jeha G, Fisheleva E, Day J, Huntsman-Labed A, Savarirayan R, Irving M, Bacino CA, Hoover-Fong J, Ozono K, Mohnike K, Wilcox WR, Horton WA, Henshaw J (2022). Pharmacokinetics and exposure-response of vosoritide in children with achondroplasia. Clin Pharmacokinet.

[102] Kaneko S, Matsushita M, Mishima K, Takegami Y, Imagama S, Kitoh H (2020). Effect of periosteal resection on longitudinal bone growth in a mouse model of achondroplasia. Bone Rep.

[103] Guojie W Evaluation of the efficacy of recombinant hum an growth hormone in the treatment of Achondroplasia[D].Xin jiang: Xinjiang Medical University,2021

